# HSP90 multi-functionality in cancer

**DOI:** 10.3389/fimmu.2024.1436973

**Published:** 2024-08-01

**Authors:** Zarema Albakova

**Affiliations:** ^1^ Department of Biology, Lomonosov Moscow State University, Moscow, Russia; ^2^ Chokan Limited Liability Partnership, Almaty, Kazakhstan

**Keywords:** HSP90, cancer, extracellular HSP90, metastasis, angiogenesis, tumor immunity

## Abstract

The 90-kDa heat shock proteins (HSP90s) are molecular chaperones essential for folding, unfolding, degradation and activity of a wide range of client proteins. HSP90s and their cognate co-chaperones are subject to various post-translational modifications, functional consequences of which are not fully understood in cancer. Intracellular and extracellular HSP90 family members (HSP90α, HSP90β, GRP94 and TRAP1) promote cancer by sustaining various hallmarks of cancer, including cell death resistance, replicative immortality, tumor immunity, angiogenesis, invasion and metastasis. Given the importance of HSP90 in tumor progression, various inhibitors and HSP90-based vaccines were developed for the treatment of cancer. Further understanding of HSP90 functions in cancer may provide new opportunities and novel therapeutic strategies for the treatment of cancer.

## 1 Introduction

Heat shock protein 90 (HSP90) chaperone machinery plays a critical role in protein folding, unfolding, degradation and maturation processes (1, 2). HSP90 chaperones interact with a large and diverse group of client proteins, many of which are important regulators of tumorigenesis, immune suppression, invasion and metastasis (3). HSP90s are primarily located in cytosol, endoplasmic reticulum, and mitochondria (4), but also have been found in the extracellular space associated with tumor progression and unfavorable clinical outcome (5). Overexpression of HSP90s has been implicated in survival and proliferation of tumor cells (6), which was further supported by the finding that HSP90s are upregulated in response to apoptotic stimuli, such as UV, sodium arsenite and doxorubicin (6–8). In addition, Kruta et al. demonstrated that *ex viv*o culture stress and aging also induce heat shock response by activating heat shock factor -1 (HSF-1) (9–11).

HSP90 family is composed of several members, including cytosolic stress-inducible HSP90α/HSP90AA1 and constitutive HSP90β/HSP90AB1, mitochondrial HSP90 called tumor necrosis factor receptor-associated protein 1 (TRAP1) and HSP90 member in endoplasmic reticulum (ER) called glucose-regulated protein 94 (GRP94/HSP90B1/gp96/ERp99/Endoplasmin) (4, 12). Different HSP90 homologs have distinct intracellular functions. For example, GRP94 is primarily responsible for the unfolded protein response whereas TRAP1 is involved in mitochondrial bioenergetics [reviewed in (12)].

In this Review, we focus on the role of HSP90 chaperone machinery in sustaining various hallmarks of cancer and exploring the potential of HSP90 as anti-cancer therapeutic targets.

## 2 The HSP90 structure and conformational cycle

**Figure 1:**
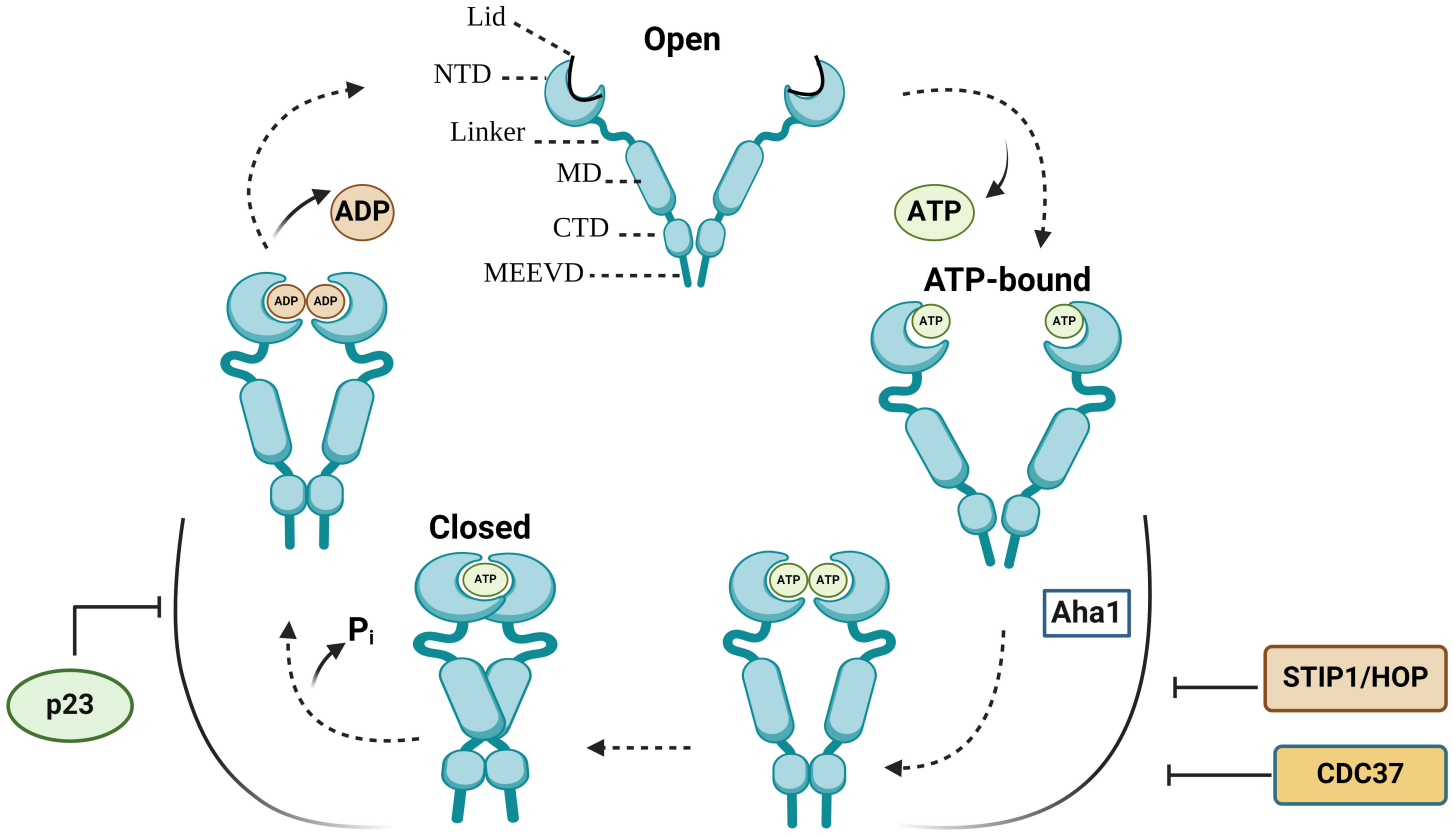
The HSP90 conformational cycle. HSP90 homodimer mainly adopts an open V-shaped conformation. ATP binding to the N-terminal domain (NTD) shifts HSP90 from an open conformation to a ‘closed and twisted’ conformation. Co-chaperones, such as p23, HSP70/HSP90-organizing protein (HOP/STIP1) and cell division cycle 37 homologue (CDC37) associate with specific HSP90 conformations. NTD, N-terminal domain; MD, middle domain; CTD, C-terminal domain; Pi, inorganic phosphate; p23, Prostaglandin E synthase 3.

Each HSP90 monomer consists of amino-terminal domain (NTD) that is connected to a middle domain (MD) by a linker, and a C-terminal domain (CTD) (**Figure 1**) (13). In the absence of ATP, HSP90 mainly adopts an open V-shaped conformation (13). ATP binding leads to the conformational change in NTD involving closure of the lids, which is followed by the NTD dimerization and twisting of HSP90 monomers for the efficient ATP hydrolysis (closed conformation) (**Figure 1**) (13–16). Various co-chaperones assist HSP90 throughout conformational cycle (13). HSP70/HSP90-organizing protein (HOP), also known as stress-inducible phosphoprotein 1 (STIP1) and cell division cycle 37 homologue (CDC37) inhibit HSP90 structural changes, whereas activator of HSP90 ATPase homologue 1 (Aha1) accelerates the formation of closed ATP-bound conformation (13, 16). Prostaglandin E synthase 3 (PTGES3/p23) acts as a co-chaperone slowing the ATPase cycle by stabilizing the closed conformation that is committed to ATP hydrolysis (13, 17) (**Figure 1**).

## 3 HSP90 post-translational modifications

One of the main challenges in studying the function of HSP90 chaperone machinery in cancer is to understand the consequences of HSP90 and co-chaperone post-translational modifications (18). Indeed, HSP90s undergo various post-translational modifications, including phosphorylation, acetylation, oxidation, ubiquitination, SUMOylation, S-nitrosylation and methylation (18). Tyrosine phosphorylation was shown to increase HSP90 interaction with endothelial nitric oxide synthase and ionotropic P2X7 receptors (19). Double-stranded DNA protein kinase (20), B-Raf (21), Akt (22), c-Src kinase (23), protein kinase A (PKA) (24), CK2 protein kinase (25, 26) have been shown to phosphorylate HSP90s, however the functional consequences of HSP90 phosphorylation are not yet fully determined (18). Kurokawa and colleagues demonstrated that by contrast to untransformed cells the HSP90β phosphorylation at Ser 226/Ser 255 was not identified in leukemic cells (26). The functions of HSP90 are also impacted by co-chaperone post-translational modifications. Several investigators showed that PP5/Ppt1 dephosphorylates Cdc37, affecting its interaction with HSP90 and its protein kinase clients (27, 28).

The chaperone activity of HSP90 is also modulated by histone deacetylase 6 (HDAC6) (18, 29–31). HDAC inhibitor depsipeptide (Romidepsin) induced acetylation of HSP90 and destabilized HSP90 interaction with several clients, including ErbB2, Raf-1, and mutant p53 in in non-small cell lung cancer cells (32). Interestingly, HDAC6 deficiency also associated with the degradation of another HSP90 client, the hypoxia-inducible factor 1α (HIF-1α) (18, 33). Additionally, HDAC6 reduction increases the acetylation of FOXP3 and HSP90, enhancing suppressive functions of T regs (34, 35). Apart from HDAC6, other HDACs are also able to deacetylate HSP90. For example, HDAC1 has been shown to deacetylate HSP90 in human breast cancer cells (36), HDAC9 in T regs (34), while both HDAC6 and HDAC10 are involved in HSP90-mediated regulation of vascular endothelial growth factor receptors (37). Thiol oxidation of HSP90 and HSP70 associates with the degradation of HSP90 client proteins, such as Cdk4, Raf-1, Akt, mutant p53 and cyclin D1 (38). Oxidative stress also causes lipid peroxidation leading to the accumulation of reactive aldehydes which in turn affect HSP90 chaperone function (12, 18, 39). HSP90 has also been reported to be ubiquitinated by CHIP (12, 40), leading to the degradation of HSP90 clients (41). In addition, S-nitrosylation, SUMOylation and methylation also affect HSP90 chaperone activity (38, 42–44).

## 4 HSP90 secretion into the extracellular milieu

Elevated HSP90 level was detected in plasma/serum in patients with cancer, including liver cancer (45), advanced staged colorectal cancer (46, 47), lung cancer (48), acute myeloid leukemia (49), hepatocellular carcinoma (50). Extracellular HSP90s may affect other cells by modulating intercellular signaling when released *via* EVs (51). EVs play important roles in intercellular communication, regulating a range of biological processes. Given the ability of EVs to carry and transfer tumorigenic factors between cells, EVs have been explored as therapeutic targets, novel drug delivery vehicles, biomarkers and standalone therapeutics in cancer research (52). HSP90s and their co-chaperones have been found in EVs isolated from patients with melanoma (53–55), glioblastoma (56), pancreatic cancer (57), prostate cancer (58), bladder cancer (59), lung cancer (60) and papillary thyroid cancer (61) [reviewed in (62)]. Lauwers and colleagues demonstrated that HSP90 in *Drosophila* regulates the membrane deformation and exosome release (63). Subsequent study demonstrated that HSP90α is located on the surface of exosomes and the monoclonal antibody against HSP90α inhibits the pro-motility activity of tumor-secreted exosomes (64).

## 5 HSP90 functions in the hallmarks of cancer

**Figure 2:**
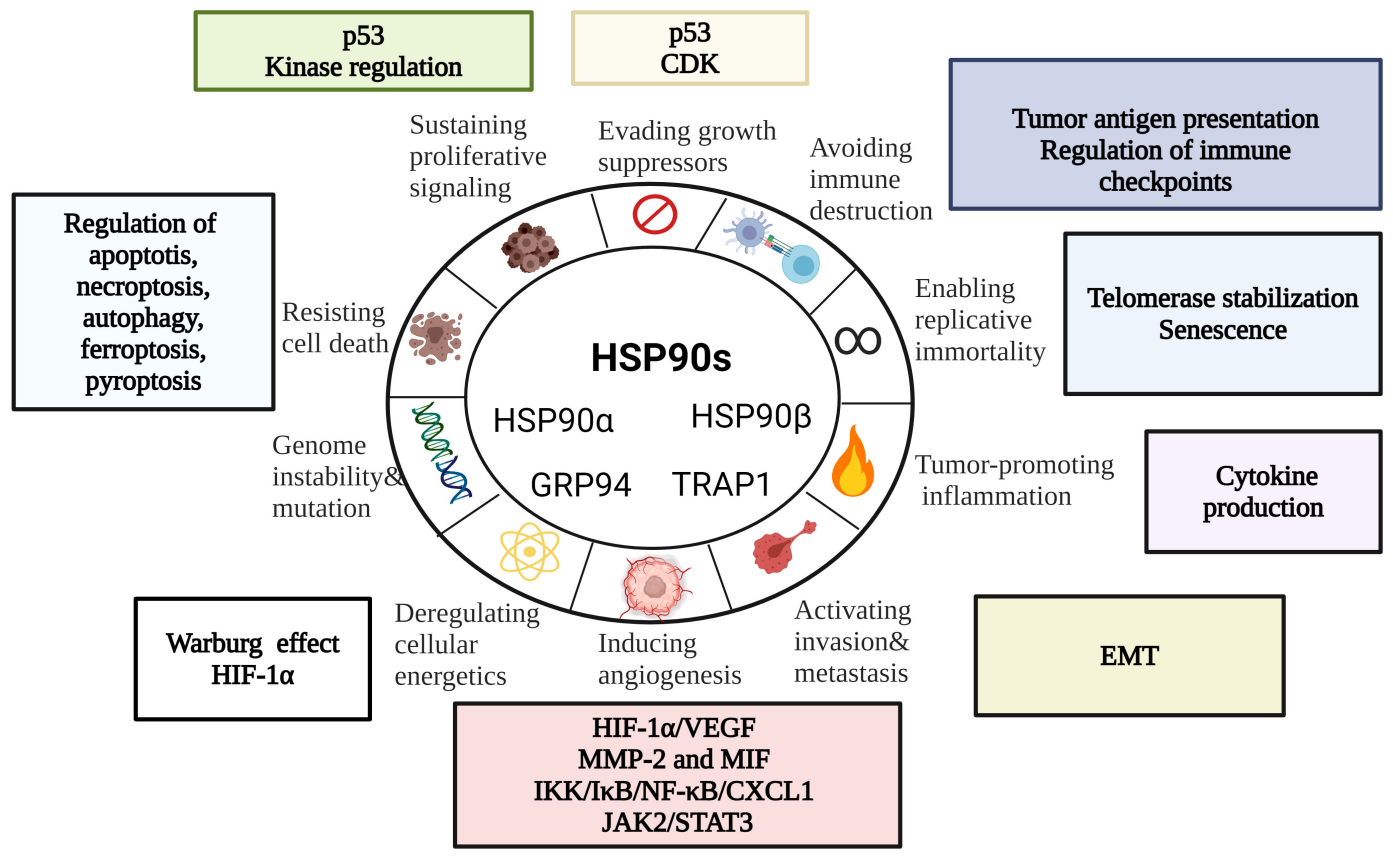
HSP90 in the Hallmarks of Cancer [modified from (67)]. HSP90s regulate cell death mechanisms, replicative immortality, tumor immunity, angiogenesis and metastasis. EMT, epithelial-mesenchymal transition; CDK, cyclin-dependent kinase; HIF-1α, hypoxia-inducible factor 1α; MIF, macrophage migration inhibitory factor; MMP-2, matrix metalloproteinase 2.

Being abundantly expressed in cancer, HSP90s promote growth and survival of tumor cells by regulating a wide range of processes. Here, we will explore HSP90 involvement in the hallmarks of cancer – a model of multi-step cancer development established by Hanahan and Weinberg (65, 66) (**Figure 2**).

### 5.1 HSP90 and tumor immunity

In 1986 Ullrich and colleagues identified HSP90 as a highly abundant cytosolic and surface tumor-transplantation antigen in methylcholanthrene-induced tumors (Meth A) (68). At the same time Srivastava et al. isolated tumor rejection antigens from the membrane and cytosol fractions of Meth A and CMS5 which was later recognized as ER HSP90 homolog, glucose-regulated protein 94 (GRP94/HSP90B1/gp96/ERp99/Endoplasmin) (69, 70). HSP90s isolated from tumors have been shown to elicit potent anti-tumor response (3, 71–73). Mechanistically, tumor-isolated HSP90-peptide complexes interact with scavenger receptor expressed by endothelial cells (SREC-I) on APCs, leading to their cross-presentation *via* MHC class I or more standard MHC class II antigen presentation pathway (2, 74). This is also supported by the finding that downregulation of heat shock factor (HSF-1) or HSP90 associates with a defective cross-presentation by DCs (75). Furthermore, it has been shown that HSP90 inhibitor reduces the translocation of antigens into the cytosol whereas *HSP90AA1* knockdown leads to a loss of proteolytic intermediates and reduced presentation of peptide-MHC I complexes on the cell surface (76, 77). Subsequent studies demonstrated that low-level inhibition of HSP90 diversifies the peptide MHC class I repertoire on tumor cells (78). HSP90 inhibitor also showed to decrease MHC II antigen presentation by IFNγ-treated APCs (79). Altogether, these data show that HSP90 is critical for MHC I and MHC II class antigen presentation.

**Figure 3:**
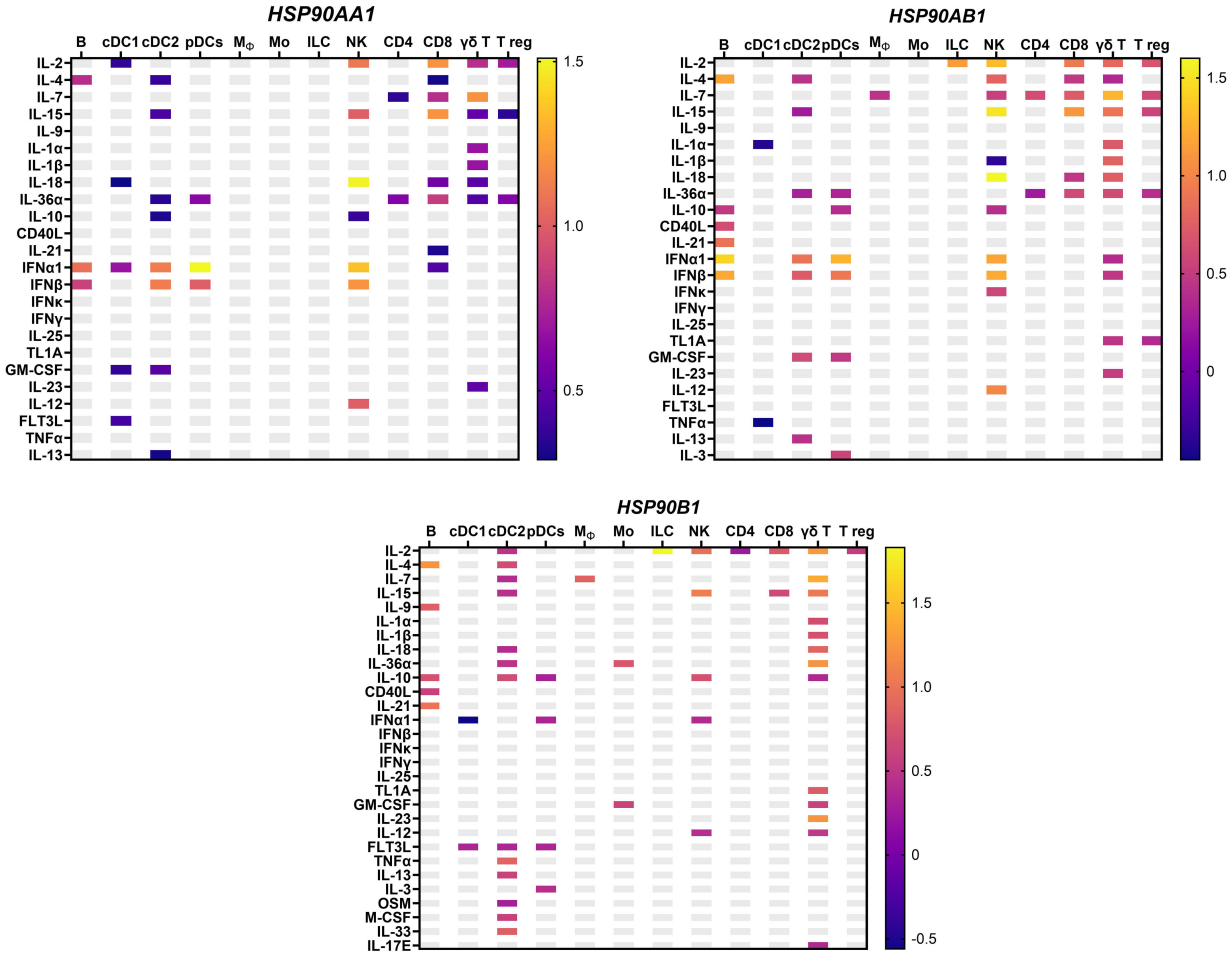
*HSP90* gene expression in response to cytokines in murine lymph nodes *in vivo* from an independent dataset (92), with the mean log2 fold change. *HSP90AA1* – cytoplasmic stress-inducible HSP90 homolog; *HSP90AB1*-cytoplasmic constitutive HSP90; *HSP90B1* – ER-resident HSP90; Mϕ, macrophages; pDC, plasmacytoid dendritic cells; B, B cell; T reg, T regulatory cells; NK, natural killer cells; Mo, monocytes; ILC, innate lymphoid cells.

Apart from antigen presentation, HSP90 is also critical for the phenotype and functional activity of immune cells. In this regard, Bae and colleagues demonstrated that HSP90 inhibitor downregulates CD3, CD8, CD25, CD28, CD40L and αβ on the surface of T cells and activating receptors, including CD2, CD11a, CD94, NKp30, NKp44, NKp46, KARp50.3 on NK cells (80). We and others show that HSP90 deficiency impairs NK and T cell proliferation, cytotoxicity and IFNγ production (80–83). By contrast, HSP90 ER homolog GRP94 stimulates NK cells indirectly *via* APCs (84). On DCs, GRP94 acts *via* Toll-like receptor 2 (TLR-2) and TLR-4 inducing the expression of CD86 and IL-12 and TNF-α production (85, 86). In T regs, GRP94 upregulates Foxp3, IL-10 and TGF-β1 *via* TLR-2/4-mediated NF-κB activation (87). Interaction of GRP94 with TLR is critical for the activation of cytotoxic T cells response (88). Additionally, GRP94 also induces NLRP3 inflammasome activation and IL-1β production in murine APCs *via* K^+^ efflux (89). HSP90α on the tumor-cell released autophagosomes (TRAPs) stimulate IL-6 release by CD4+ T cells *via* TLR2-MyD88-NF-κB pathway (90). Autocrine IL-6 further promotes the production of IL-10 and IL-21 by CD4^+^ T cells *via* STAT3, enhancing metastasis (90). It has also been shown that the production of HSP90α, IL-8 and IL-6 by macrophages induces JAK2-STAT3 pathway, supporting invasion and migration in pancreatic ductal epithelial cells (91). On the other hand, cytokines may also induce *HSP90* expression, which further enhance their pro and anti-inflammatory activities (**Figure 3**) (92). Unlike *HSP90AA1, HSP90AB1* and *HSP90B1, TRAP1* could only be induced by IL-18 in NK cells and IL-3 in conventional DC2 (cDC2) cells (92). Collectively, these studies show that there is an important interplay between HSP90 and cytokines, which should be further explored in the context of cancer.

HSP90 family members also play important roles in the regulation of immune checkpoints. Zavareh and colleagues demonstrated that HSP90 inhibitors downregulate PD-L1 mRNA level and surface expression by suppressing HSP90 clients c-Myc and signal transducer and activator of transcription 3 (STAT3) (93). Another HSP90 client nucleophosmin/anaplastic lymphoma kinase (NPM/ALK) showed to induce PD-L1 *via* STAT3 activation in T cell lymphoma cells (94). It has been also shown that the spliced isoform of HSP90 co-chaperone FKBP51 regulates the expression of glycosylated PD-L1 in glioma cells (95). Combination of HSP90 inhibitor ganetespib and anti-CTLA-4 associated with an increase in the frequency of CD8^+^ T cells in mice and decrease in T regs (96). Mechanistically, HSP90 inhibitor upregulates interferon response genes, leading to T cell-mediated killing of melanoma cells (96).

**Figure 4:**
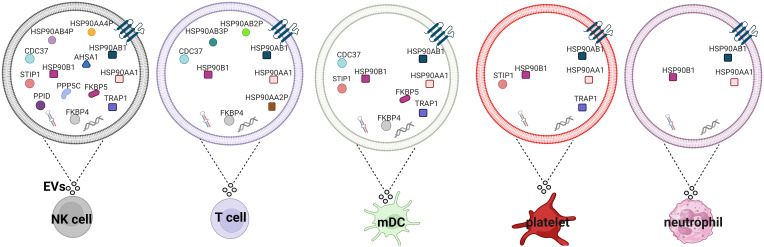
HSP90s and their co-chaperones in extracellular vesicles secreted by different types of immune cells. Extracellular vesicles secreted by NK cells (97), T cells (98), mDCs (99), platelets (100) and neutrophils (101) isolated from healthy donors. EVs, extracellular vesicles; mDCs, monocyte-derived dendritic cells.

Using mass spectrometry-based proteome profiling several studies showed that various types of immune cells, including NK, T, dendritic cells, platelets, and neutrophils can secrete HSP90s and their cognate co-chaperones in EVs (summarized in **Figure 4**) (62). Overexpression of HSP90 in hypoxic macrophage-derived exosomes inhibited Hippo signaling pathway, leading to colorectal cancer progression (102). Heat shock and anti-cancer drugs significantly upregulate exosomes release (103). Exosomes secreted by mouse B cell lymphoma cells after heat shock showed elevated expression of HSP90, HSP60 and MHC I, MHC II, CD40, CD86, RANTES and IL-1β (104, 105). These exosomes stimulate DC maturation and more potently induce CTL responses (104). It has also been shown that HSP-bearing exosomes secreted by human hepatocellular carcinoma cells stimulate NK cell cytotoxicity and granzyme B secretion (103). Triple deletion of CDC37, HSP90α and HSP90β diminished EV-driven malignancy progression and macrophage M2 polarization (106).

### 5.2 HSP90 in tumor resistance to cell death

HSP90 regulates both intrinsic and extrinsic apoptotic pathways. In intrinsic pathway, HSP90 is implicated in the conformational change of Bax and the release of cytochrome *c* (107, 108). Moreover, HSP90 also interacts with Apaf-1, inhibiting pro-caspase-9 and pro-caspase-3 activation (6). HSP90 inhibition downregulates STAT3, survivin, cyclin D1 and upregulates cytochrome *c*, caspase-9 and caspase-3 (109). Results also showed that TRAP1 inhibitor gamitrinib containing triphenylphosphine induces cyclophilin D-dependent mitochondrial permeability transition in tumor cells, leading to apoptosis (108, 110, 111). In extrinsic pathway, FLICE-like inhibitory proteins (c-FLIP) is required for inhibiting apoptosis at the death inducing signaling complex (DISC) (108, 112). HSP90 inhibitors induced c-FLIP_L_ degradation in human lung cancer cells mediated by C-terminus of HSP70-interacting protein (CHIP) (112).

HSP90 is also involved in the modulation of another form of regulated cell death necroptosis (108, 113). Jacobsen and co-workers demonstrated that HSP90 inhibitors block necroptosis by downregulating MLKL expression and membrane translocation (113). Several studies reported that HSP90 inhibitors impact RIP1 stability and function (114–117). A complex consisting of HSP90 and CDC37 is required for RIP3 activation during necroptosis (118).

Apart from apoptosis and necroptosis, HSP90 is implicated in autophagy. HSP90 is essential for the lysosome-associated membrane protein type 2A (LAMP-2A) stability (119). Moreover, HSP90 inhibition leads to the IκB kinase (IKK) degradation by autophagy while Atg5 or autophagy inhibition can reverse IKK degradation, suggesting that there is a molecular link between HSP90, NF-κB and autophagy (108, 120). In addition, HSP90/CDC37 stabilizes and activates ULK1, which is required for Atg13 phosphorylation and release. Subsequently, Atg13 is recruited to damaged mitochondria for efficient clearance (121). HSP90 inhibition downregulates Atg7 and upregulates caspase 9 in *KRAS*- mutant non-small cell lung cancer cells (122). HSP90 inhibition also leads to Beclin 1 proteasomal degradation, suppressing TLR3- and TLR4-mediated autophagy (123).

In addition, HSP90 is involved in ferroptosis facilitating the degradation of glutathione peroxidase 4 (GPX4) by chaperone-mediated autophagy (117, 124). It is interesting to note that HSP90 inhibitor 2-amino-5-chloro-N,3-dimethylbenzamide (CDDO) can block both necroptosis and ferroptosis, suggesting that HSP90 may be a common regulatory mechanism in necroptosis and ferroptosis (117). HSP90 is also implicated in pyroptosis by regulating priming and activation of NLRP3 inflammasome and subsequent IL-1β production (125–127).

### 5.3 HSP90 in sustained proliferation

Recent studies have reported that HSP90 regulates the activity of tumor suppressor p53 by interacting with its DNA binding domain (128). HSP90 stabilizes mutant p53 in cancer cells leading to uncontrolled proliferation of tumor cells (129, 130). HSP90 also stabilizes the epidermal growth factor receptor (EGFR) in tumor cells (129). HSP90 inhibition decreases total and phosphorylated EGFR and suppresses the proliferation of resistant cancer cells (131). In addition, HSP90 activity is essential for ErbB2/HER, v-Src, c-Src, BCR-ABL, Raf1, and other kinases which are known to promote proliferation and survival of cancer cells (132).

### 5.4 HSP90 in the deregulation of cellular energetics

HSP90 homolog TRAP1 is a critical regulator of mitochondrial bioenergetics (12). TRAP1 interacts and suppresses the activity of succinate dehydrogenase (SDH), promoting Warburg phenotype (133). Results also showed that TRAP1 decreases cell oxygen consumption rate and OXPHOS-dependent ATP synthesis (133). Furthermore, *TRAP1* deficiency enhances mitochondrial respiration and inhibits glycolysis (134). These TRAP1-deficient cells also express increased levels of ATP, ROS and cytochrome *c* oxidase (complex IV) (134). Mitochondrial HSP90 homolog TRAP1, but not cytosolic HSP90, binds and stabilizes succinate dehydrogenase-B (SDHB) contributing to HIF-1α-mediated cancer progression in patients carrying *SDHB* mutations (135).

### 5.5 HSP90 in replicative immortality

Holt and colleagues demonstrated that HSP90 and its co-chaperone p23 associate with human telomerase reverse transcriptase and are required for efficient assembly of functional telomerase (136). HSP90 inhibitor geldanamycin inhibited the assembly of active telomerase *in vitro* and *in vivo* (136). Further biochemical studies demonstrated that HSP90 is critical for hTERT folding and stabilization of the assembled telomerase complex (137). HSP90 is also important for the maintenance of telomere length as overexpression of HSP90 associates with telomere shortening (138). In addition, HSP90 promotes telomerase DNA binding (139). Telomere dysfunction may also induce senescence (140). Indeed, Zhong and colleagues demonstrated that an increase in extracellular HSP90α promotes fibroblast senescence by activating TGFβ (141). HSP90 inhibitors downregulate phosphorylated form of AKT, leading to apoptosis of senescent cells (142). These data suggest that HSP90 favors tumor growth by modulating telomerase and senescence.

### 5.6 HSP90 in angiogenesis

Song and colleagues reported that HSP90α promotes angiogenesis *via* stabilizing activated matrix metalloproteinase 2 (MMP-2) (143). Further studies showed that HSP90 also stabilizes macrophage migration inhibitory factor (MIF), which acts as an angiogenesis promoting factor during neoplastic transformation (144, 145). Dong et al. demonstrated that breast cancer cells secrete HSP90α to survive under hypoxia (146). HSP90 inhibitor AT-533 has been reported to inhibit growth and angiogenesis by suppressing the HIF-1α/VEGF pathway in hypoxic breast cancer cells (147). These cells also secrete a splice variant VEGF_90K_ which binds HSP90 on the surface of microvesicles further promoting angiogenesis (148). HSP90/phosphorylated IKK-rich extracellular vesicles from hypoxic melanoma activate pro-angiogenic melanoma-associated fibroblasts (MAFs) *via* the NF-κB/CXCL1 axis (149). Furthermore, C-terminal HSP90 inhibitor SL-145 has been shown to inhibit growth and angiogenesis by dysregulating JAK2/STAT3 signaling pathway in triple negative breast cancer cells (150).

### 5.7 HSP90 in invasion and metastasis

Extracellular HSP90 interacts with LRP1 (also known as CD91) to induce ERK and MMP-2/9 activation, leading to E-cadherin inhibition and the initiation of EMT in prostate cancer cells (51, 151). Furthermore, extracellular HSP90 secreted by these cells upregulates the expression of stem-like markers, promoting self-renewal (152). HSP90 interaction with LRP1 leads to the increased expression of phosphorylated IKKα/β and NF-κB resulting in the induction of TCF12, which in turn decreases E-cadherin and promotes colorectal cancer EMT, migration and invasion (153). HSP90β also associates with LRP5, promoting EMT *via* Akt and Wnt/β-catenin signaling (12, 154). In metastatic breast cancer cells, HIF-1α downregulation inhibits HSP90α secretion and invasion (155). GRP94, an ER paralog of HSP90 may also promote invasion and metastasis *via* the regulation of its client GARP, which is critical for the membrane expression of TGFβ (156).

## 6 HSP90 therapies targeting cancer

### 6.1 HSP90 inhibitors in cancer clinical trials

Owing to the importance of HSP90 in cancer, it has become an attractive target for anti-cancer therapies. HSP90 inhibitors in clinical trials are summarized in **Table 1**. Several clinical trials assessed HSP90 inhibitor-linked to verteporfin (HS-201, NCT03906643) or near infrared red probe (HS-196, NCT03333031) for imaging and detection of solid tumors. Currently, there are no FDA-approved HSP90 inhibitors. The low effectiveness of HSP90 inhibitors in clinical trials may be attributed to drug-related toxicity and limited efficacy. Insufficient isoform selectivity has been considered as one of the main reasons for these failures.

**Table 1:** HSP90 inhibitors in cancer clinical trials.

HSP90 inhibitors	Types of cancer	Clinical trial phase
MPC-3100	Refractory or relapsed cancer	Phase I; NCT00920205
Gamitrinib	Advanced cancer	Phase I; NCT04827810
AUY922	Advanced solid tumors	Phase I; NCT01602627
AUY922	Refractory or recurrent lymphoma	Phase II; NCT01485536
AUY922+ Capecitabine	Advanced solid tumors	Phase I; NCT01226732
AUY922+ Pemetrexed Disodium;	Stage IV NSCLC	Phase I; NCT01784640
AUY922+ Erlotinib Hydrochloride	Stage IIIB-IV NSCLC	Phase I/II; NCT01259089
AUY922+BYL719	Advanced or metastatic gastric cancer with PIK3CA alteration or HER2 amplification	Phase Ib; NCT01613950
AUY922	Refractory gastrointestinal stromal tumor	Phase II; NCT01404650
AUY922	Myelofibrosis, essential thrombocythemia, polycythemia vera	Phase II; NCT01668173
AUY922	Advanced NSCLC	Phase II; NCT01124864
AUY922	Advanced ALK-positive NSCLC	Phase II; NCT01752400
SNX-5422	Refractory solid tumors; lymphoma	Phase I; NCT00647764
AT13387 (Onalespib) + Talazoparib	Recurrent ovarian, fallopian tube, peritoneal cancer or recurrent triple-negative breast cancer	Phase I; NCT02627430
AT13387 or ATT13387+ Abiraterone Acetate	Castration-resistant prostate cancer	Phase I/II; NCT01685268
AT13387+ AT7519M	Advanced solid tumors	Phase I; NCT02503709
AT13387 or AT13387+ Crizotinib;	NSCLC	Phase I/II; NCT01712217
AT13387	Refractory solid tumors	Phase I; NCT01246102
AT13387+ Paclitaxel	Advanced, triple negative breast cancer	Phase Ib; NCT02474173
AT13387+ Olaparib	Advanced solid tumors	Phase I; NCT02898207
AT13387	Anaplastic large cell lymphoma, mantle cell lymphoma, diffuse large B-cell lymphoma	Phase II; NCT02572453
KW-2478+Bortezomib	Relapsed or refractory multiple myeloma	Phase I/II; NCT01063907
Ganetespib (STA-9090)	Stage I-IVA squamous cell carcinoma of the head and neck	Phase I; NCT02334319
Ganetespib + Paclitaxel	Recurrent, platinum-resistant ovarian, fallopian tube or primary peritoneal cancer	Phase I/II; NCT01962948
Ganetespib	Relapsed or refractory small cell lung cancer	Phase II;NCT01173523
Ganetespib	Metastatic hormone-resistant prostate cancer previously treated with docetaxel-based chemotherapy	Phase II; NCT01270880
Ganetespib	Metastatic ocular melanoma	Phase II; NCT01200238
Ganetespib	Hematologic malignancies	Phase I; NCT00858572
Ganetespib	Solid tumors	Phase I; NCT00687934
Ganetespib	HER2+ or triple negative breast cancer	Phase II; NCT01677455
Ganetespib	Metastatic pancreas cancer	Phase II; NCT01227018
Ganetespib+Paclitaxel+ Trastuzumab + Pertuzumab	Human epidermal growth factor receptor 2- metastatic breast cancer	Phase I; NCT02060253
Ganetespib	Acute myeloid leukemia, acute lymphoblastic leukemia, blast-phase chronic myelogenous leukemia	Phase I; NCT00964873
Ganetespib+ Paclitaxel, Carboplatin +radiation therapy	Stage II-III patients with esophageal carcinoma	Phase I; NCT02389751
Ganetespib	Stage III or Stage IV melanoma	Phase II; NCT01551693
Ganetespib	Epithelial ovarian cancer	Phase I/II; NCT02012192
Ganetespib+ Sirolimus	Malignant peripheral nerve sheath tumors	Phase I/II; NCT02008877
Ganetespib+ Ziv-Aflibercept	Refractory gastrointestinal carcinomas, non-squamous NSCLC, urothelial carcinomas, sarcomas	Phase I; NCT02192541
Ganetespib+ Docetaxel	Solid tumors	Phase I; NCT01183364
Ganetespib	Stage IIIB or IV NSCLC	Phase II; NCT01031225
HS-201	Solid tumors	Phase I; NCT03906643
XL888+Vemurafenib+Cobimetinib;	Unresectable BRAF-mutated stage III/​IV melanoma	Phase I; NCT02721459
XL888+ Pembrolizumab	Advanced gastrointestinal tumors	Phase Ib; NCT03095781
XL888+Vemurafenib	Unresectable BRAF- mutated stage III/IV Melanoma	Phase I; NCT01657591
PU-H71 + Ruxolitinib	Primary myelofibrosis, post-polycythemia vera myelofibrosis, post-essential thrombocythemia myelofibrosis	Phase Ib; NCT03935555
PU-H71	Refractory solid tumors and low-grade non-Hodgkin’s lymphoma	Phase I; NCT01581541
IPI-504	NSCLC with ALK translocations;	Phase II; NCT01228435
IPI-504	Relapsed/​refractory Stage IIIb, or Stage IV NSCLC or Stage IV NSCLC	Phase I/II; NCT00431015
IPI-504	Gastrointestinal stromal tumors	Phase III; NCT00688766
IPI-504	Advanced breast cancer	Phase II; NCT00627627
IPI-504	Metastatic melanoma	Phase II; NCT00627419
HS-196	Solid tumors	Phase I; NCT03333031
BIIB021 (CNF2024)	B-cell chronic lymphocytic leukemia	Phase I; NCT00344786
BIIB021 (CNF2024)	Advanced solid tumors	Phase I; NCT00345189
17-DMAG (Alvespimycin)	Relapsed chronic lymphocytic leukemia/small lymphocytic lymphoma, B-cell prolymphocytic leukemia	Phase I; NCT01126502
DS-2248	Advanced solid tumors	Phase I; NCT01288430
TAS-116 (Pimetespib) + Palbociclib	Advanced breast cancer	Phase Ib; NCT05655598
TAS-116	Solid tumors	Phase I; NCT02965885
SNX-5422	Refractory solid tumors, lymphomas	Phase I; NCT00644072
Debio 0932 + chemotherapy	Stage IIIb or IV NSCLC	Phase I; NCT01714037
CNF1010 (lipid formulation of 17-AAG)	ZAP-70^+^ B-Cell Chronic Lymphocytic Leukemia	Phase I; NCT00319930

### 6.2 HSP90 vaccines

The ability of HSP90-peptide complexes to activate both CD8^+^ and CD4 ^+^T cells led to the development of HSP90-based vaccines (3, 157). Innovative approach was proposed by Yamazaki and colleagues who generated a secretory form of ER HSP90 where HSP90 ER (gp96) KDEL retention signal was deleted and replaced with the Fc portion of IgG1, thus imitating necrotic cell death release of HSPs (158). Immunization of mice with tumor cells secreting gp96-Ig resulted in tumor rejection *in vivo* which was primarily dependent on CD8^+^ T cells (158, 159). Gp96-Ig vaccine, also called Viagenpumatucel-L or HS-110 was further assessed in phase I (NCT00503568) and phase II (NCT02117024) clinical trials in patients with non-small cell lung carcinoma. Gp96-Ig was also assessed in combination with anti-PD-1inhibitor Nivolumab (NCT02439450) and has shown to be well-tolerated and improve overall survival of PD-L1^+^ patients with advanced lung cancer (160, 161).

Crane and colleagues prepared autologous gp96-peptide complexes to immunize patients with recurrent glioblastoma in phase I trial (162). Re-stimulation of peripheral blood leukocytes with autologous gp96 led to increase in IFNγ (162). Autologous gp96 prepared from resected tumors in combination with standard radiation and chemotherapy improved overall survival in glioblastoma patients with low expression of PD-L1^+^ on peripheral myeloid CD45^+^ CD11b^+^ cells (163). Interestingly, dendritic cells (DCs) pulsed with tumor-derived gp96 showed anti-tumor effect which was significantly dependent on NK and CD8 T cells (164). Multi-chaperone vaccine called “chaperone-rich cell lysate” (CRCL) contains several chaperones, including HSP70, HSP90, gp96 and calreticulin showed to activate DCs and upregulate the expression of CD40, MHC II, IL-12, CD70, iNOS and NF-κB and enhance the phosphorylation of STAT1,STAT5, ERK1/2 and AKT (165, 166). CRCL-stimulated DCs and macrophages resisted the suppressive activity of T regulatory cells (167). Notably, depletion of chaperones from CRCL led to the decrease in IFNγ production by splenocytes (165). Similar to T cells, CRCL has also been shown to stimulate IFNγ, TNFα, RANTES production and the activation of STAT1 and NF-κB by NK cells (168).

Immunization of mice with another multi-chaperone vaccine purified from the mouse sarcoma cell line S180 containing the mixture of HSP60, HSP70, HSP110 and gp96 (mHSP/peptide vaccine) in combination with cyclophosphamide and IL-12 suppressed tumor growth and improved long-term survival (169). Further studies have shown that mHSP/peptide vaccine containing HSP70, HSP90 and gp96 showed superior anti-tumor effect than gp96/peptide vaccine (170). PD-L1 inhibitor in combination with tumor-derived mHSP/peptide vaccine induced the section of IFNγ, TNFα, IL-10 and IL-2 on day 14^th^ whereas on day 28^th^ combinational treatment led to decrease production of IFNγ, IL-2 and IL-10 (170).

## 7 Conclusion

HSP90 molecular chaperones are abundantly expressed in cancer, leading to tumor growth and survival *via* the modulation of various hallmarks of cancer, including sustained proliferation, deregulation of cellular energetics, unlimited replicative potential, tumor immunity, angiogenesis, metastasis and invasion. Given HSP90 ability to promote growth and survival of tumor cells by regulating a wide range of processes and enabling hallmarks of cancer, various HSP90 inhibitors entered clinical trials. Based on the ability of HSP90 to elicit anti-tumor response, several HSP90-based immunotherapies were developed. Further elucidating the complex role of HSP90 in cancer may provide new opportunities for the diagnosis and treatment of cancer patients.

## Author contributions

ZA: Writing – review & editing, Writing – original draft, Visualization, Resources, Project administration, Investigation, Conceptualization.
